# (*E*)-2-Cyano-3-(2,3-dimeth­oxy­phen­yl)acrylic acid

**DOI:** 10.1107/S1600536811051129

**Published:** 2011-11-30

**Authors:** Aliakbar Dehno Khalaji, Karla Fejfarová, Michal Dušek

**Affiliations:** a Department of Chemistry, Faculty of Science, Golestan University, Gorgan, Iran; b Institute of Physics of the ASCR, v.v.i, Na Slovance 2, 182 21 Praha 8, Czech Republic

## Abstract

The asymmetric unit of the title compound, C_12_H_11_NO_4_, contains two mol­ecules. In the crystal, neighbouring mol­ecules are linked together by O—H⋯O hydrogen bonds into dimers. The dimers are arranged into columns parallel to the *a* axis, meditated by π–π inter­actions [centroid–centroid distances = 3.856 (3) and 3.857 (3) Å]. The crystal structure is further stabilized by weak inter­molecular C—H⋯O inter­actions. The crystal studied was a non-merohedral twin with a ratio of the twin components of 0.657 (11):0.343 (11).

## Related literature

For applications of cyano­acrylic acid derivatives, see: Hagberg *et al.* (2006 ▶); Kim *et al.* (2008 ▶); Hara *et al.* (2003 ▶). For structures and properties of complexes based on carboxyl­ate ligands, see, for example: Zhao *et al.* (2008 ▶); Wang *et al.* (2009 ▶); Mitra *et al.* (2006 ▶); Shit *et al.* (2009 ▶); Akhbari *et al.* (2009 ▶).
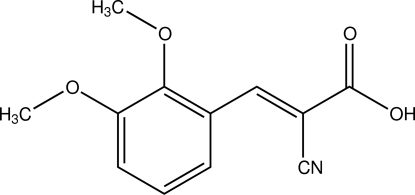


## Experimental

### 

#### Crystal data

C_12_H_11_NO_4_
*M* *_r_* = 233.2
Monoclinic, 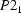

*a* = 3.8564 (5) Å
*b* = 27.178 (3) Å
*c* = 10.4681 (9) Å
β = 99.966 (9)°
*V* = 1080.6 (2) Å^3^
*Z* = 4
Cu *K*α radiation
μ = 0.92 mm^−1^
*T* = 120 K
0.57 × 0.15 × 0.05 mm

#### Data collection

Agilent Xcalibur diffractometer with an Atlas (Gemini ultra Cu) detector
Absorption correction: multi-scan (*CrysAlis PRO*; Agilent, 2010 ▶) *T*_min_ = 0.525, *T*_max_ = 1
17777 measured reflections
1922 independent reflections
1638 reflections with *I* > 3σ(*I*)
*R*_int_ = 0.083

#### Refinement

*R*[*F* ^2^ > 3σ(*F* ^2^)] = 0.049
*wR*(*F* ^2^) = 0.129
*S* = 1.73
1922 reflections
314 parameters
2 restraints
H atoms treated by a mixture of independent and constrained refinement
Δρ_max_ = 0.23 e Å^−3^
Δρ_min_ = −0.23 e Å^−3^

### 

Data collection: *CrysAlis PRO* (Agilent, 2010 ▶); cell refinement: *CrysAlis PRO*; data reduction: *CrysAlis PRO*; program(s) used to solve structure: *SIR2002* (Burla *et al.*, 2003 ▶); program(s) used to refine structure: *JANA2006* (Petříček *et al.*, 2006 ▶); molecular graphics: *DIAMOND* (Brandenburg & Putz, 2005 ▶) and *COOT* (Emsley *et al.*, 2010 ▶); software used to prepare material for publication: *JANA2006*.

## Supplementary Material

Crystal structure: contains datablock(s) global, I. DOI: 10.1107/S1600536811051129/bt5719sup1.cif

Structure factors: contains datablock(s) I. DOI: 10.1107/S1600536811051129/bt5719Isup2.hkl

Supplementary material file. DOI: 10.1107/S1600536811051129/bt5719Isup3.cdx

Supplementary material file. DOI: 10.1107/S1600536811051129/bt5719Isup4.cml

Additional supplementary materials:  crystallographic information; 3D view; checkCIF report

## Figures and Tables

**Table 1:** Hydrogen-bond geometry (Å, °)

*D*—H⋯*A*	*D*—H	H⋯*A*	*D*⋯*A*	*D*—H⋯*A*
O6—H6*o*⋯O1^i^	0.85 (5)	1.82 (6)	2.62 (3)	154 (6)
O2—H2*o*⋯O5^ii^	0.85 (6)	1.79 (6)	2.63 (3)	169 (8)
C11—H11*c*⋯O4^iii^	0.96	2.56	3.50 (3)	167
C23—H23*a*⋯O3^iii^	0.96	2.56	3.42 (4)	149

Symmetry codes: (i) 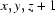; (ii) 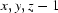; (iii) 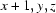.

**Fig. 1.**
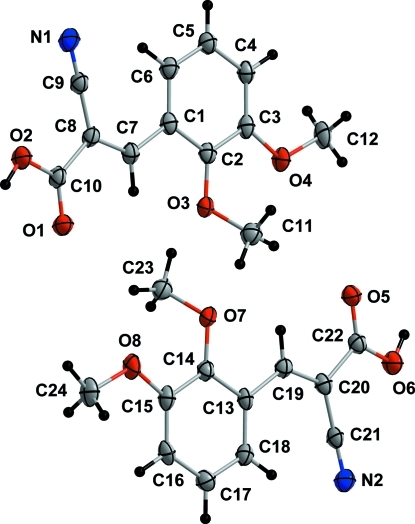
The structure of the title compound. Displacement ellipsoids are drawn at the 50% probability level.

**Fig. 2.**
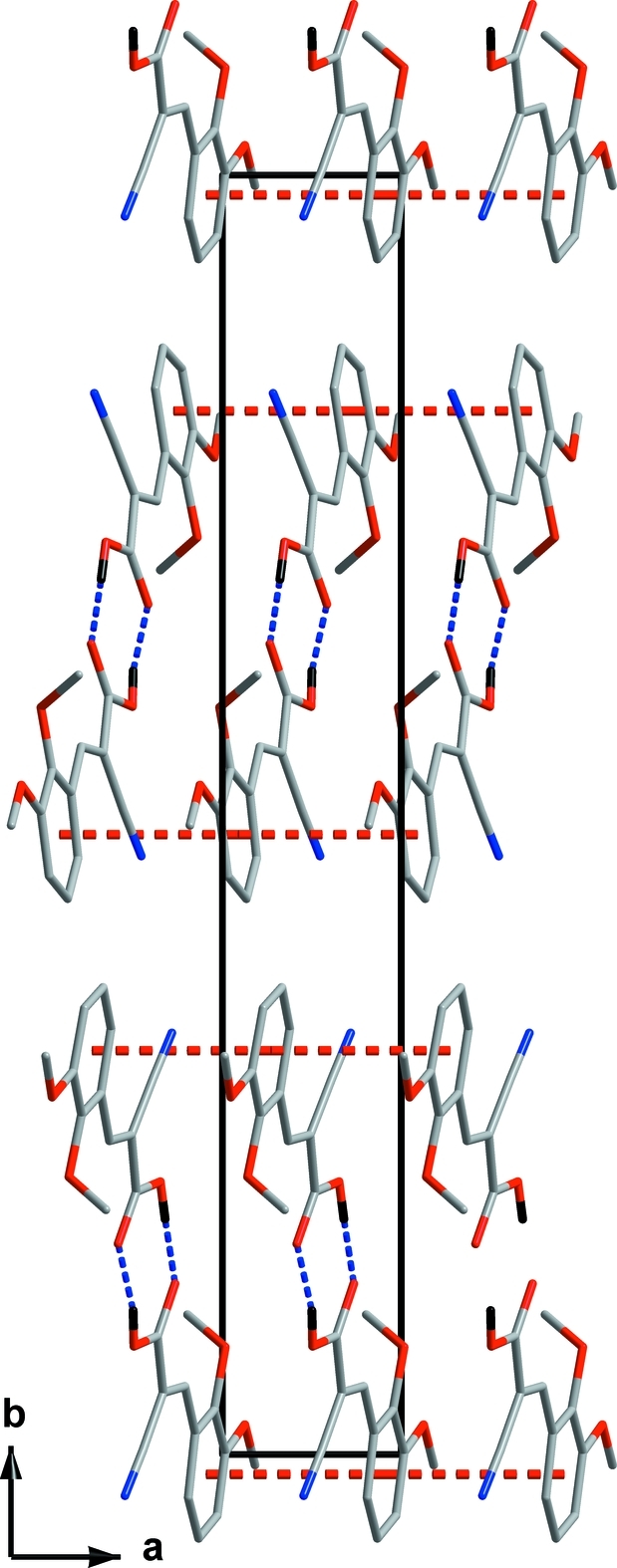
Crystal packing of the title compound, viewed along the c axis. Hydrogen bonds are drawn as blue dashed lines, π-π interactions as red dashed lines. Hydrogen atoms not participating in hydrogen bonds were omitted for clarity.

**Fig. 3.**
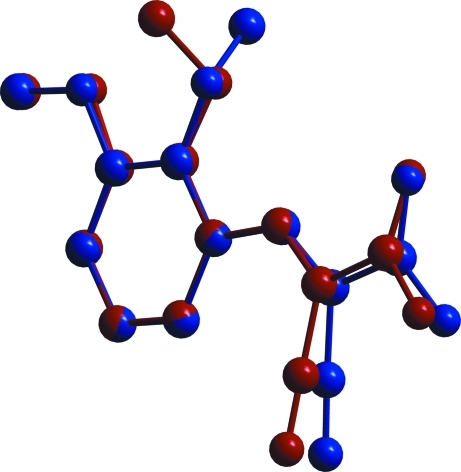
Overlay of two molecules present in asymmetric unit. Red: C1—C12, N1, O1–O4, blue: C13–C24, N2, O5–O7. Hydrogen atoms are omitted for clarity.

**Fig. 4.**
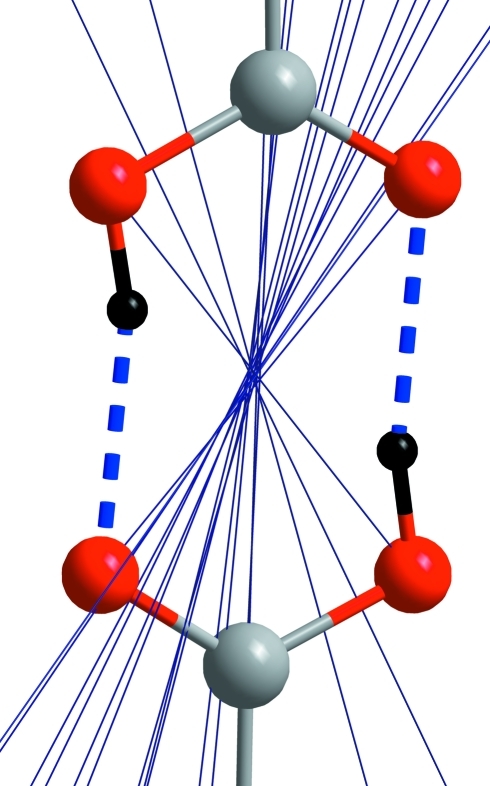
Visual inspection of a possible non-crystallographic inversion center. The lines are connecting corresponding atoms of the two molecules.

## supplementary crystallographic information

### Comment

Carboxylate compounds, an important family of O-donor ligands, can coordinate to different metal ions forming versatile structures with different topologies and stabilities (Zhao *et al.*, 2008; Wang *et al.*, 2009). Transition metal complexes with these ligands have special properties and applications (Mitra *et al.*, 2006; Shit *et al.*, 2009; Akhbari *et al.*, 2009;). Among the carboxylate compounds, cyanoacrylic acid derivatives are of great importance to convert solar light into electricity in dye-sensitized solar cells (Hagberg *et al.*, 2006; Kim *et al.*, 2008 and Hara *et al.*, 2003).

In consideration of the important applications of carboxylate compounds, herein the crystal structure of the title compound, (I), is reported. Systematic characterization of (I) has been performed by elemental analyses, FT—IR, ^1^H-NMR, UV-Vis spectroscopy and *x*-ray crystallography

The molecular structure of (I) with the atom-numbering scheme is presented in Fig. 1. The asymmetric unit of (I) contains two molecules, which differ mainly in the orientation of methoxy groups (Fig. 3) as reflected in the C1—C2—O3—C11 and C13—C14—O7—C23 torsion angles of -120.5 (7)° and 121.0 (7) °, respectively. In both molecules, the phenyl ring and the chain connecting the ring to the CN and COOH groups are nearly coplanar. The dihedral angles between the planes of the phenyl rings (C1–C6, C13–C18) and the planes defined by C7–C10 and C19–C22, are 5.7 (4)° and 5.5 (4) °, respectively. This degree of coplanarity allows for increased π-conjugation in the title compound.

Careful inspection of the packing diagram (Fig. 2) lead us to assumption that the two independent molecules could be related by non-crystallographic inversion center. To test this assumption, we used in the first step a rigid-body approach available in the crystallographic package *JANA2006* (Petříček *et al.*, 2006). The atoms of the first molecule (C1—C12, N1, O1–O4) were taken as a model molecule and refined to obtain the geometry of the model molecule. Together with the model molecule we refined a translation vector and three rotation angles transforming the model molecule to the actual position *A* (C1—C12, N1, O1–O4) and another translation vector and three rotation angles transforming the inverted model molecule to the actual position *B* (C13–C24, N2, O5–O7). The resulting *R* value was 5.2% for 164 refined parameters, providing evidence that the geometry of *A* and inverted geometry of *B* can be considered identical. In the second step, we graphically connected corresponding atoms in the two identical molecules *A* and *B* by lines (Fig. 4) to inspect visually position of the possible non-crystallographic inversion centre. The lines did not intersect at the same point but the obtained intersections were very close indicating that the inversion center is present only approximately. In order to quantify this finding in terms of *R* value we used the original structure model without rigid body (*i.e.* the one reported in this article) and we restricted the coordinates and ADP parameters of corresponding atoms of the two molecules by a local inversion symmetry operation located at the average position of the intersections shown in Fig. 4. Indeed, the resulting *R* value increased to 7.1% for 158 refined parameters.

In the crystal, the molecules form hydrogen-bonded dimers *via* the carboxyl groups. These dimers are connected by π-π interactions (centroid-centroid distances 3.856 (3), 3.857 (3) Å into columns parallel to the *a* axis (Fig. 2). The crystal structure is further stabilized by weak intermolecular C—H···O interactions.

The crystal studied was a non-merohedral twin with the ratio of the twin components of 0.657 (11):0.343 (11).

### Experimental

2,3-dimethoxybenzaldehyde (0.4 mmol) and cyanoacetic acid (0.4 mmol) were dissolved in a mixture of methanol:acetonitrile (1:1 *v/v*, 20 ml) in the presence of piperidine (0.4 mmol). The mixture was stirred and refluxed for 1.5 h to give a clear yellow solution. The mixture was cooled and the product was allowed to crystallize by slow evaporation technique at room temperature. After 5 days, yellow precipitate of (I) was formed which was collected by filtration and dried at room temperature. Recrystallization of the yellow precipitate from acetonitrile-chloroform mixture (2:1 *v/v*, 30 ml) by adding acetic acid afforded yellow crystals of 2-cyano-3-(2,5-dimethoxyphenyl)acrylic acid (1). Yield: 91%. *Anal*. Calc. for C_12_H_11_NO_4_: C, 61.80; H, 4.75; N, 6.01%. Found: C, 61.77; H, 4.79; N, 6.08%. FT—IR (KBr, cm^-1^): 2922–3059, 2839, 2220, 1697, 1678, 1571, 1497, 1469, 1458, 1425, 1381, 1364, 1297, 1248, 1234. UV–Vis, λ_max_ (nm)/ε (*M*^-1^cm^-1^); 241 (9287), 310 (25074). ^1^H NMR (DMSO-*d_6_*): *δ* = 3.81 (s, 3H), 3.85 (s, 3H), 7.25 (t, 1H), 7.33 (dd, 1H), 7.71 (dd, 1H), 8.47 (s, 1H), 14.08 (b, 1H) p.p.m..

### Refinement

All H atoms bonded to carbon atoms were positioned geometrically and treated as riding on their parent atoms. The methyl H atoms were allowed to rotate freely about the adjacent C—O bonds. The carboxyl H atoms were found in difference Fourier maps and their coordinates were refined with restraint on the O—H bond length 0.85 Å with σ of 0.02. All H atoms were refined with   displacement coefficients *U*_iso_(H) set to 1.5*U*_eq_(C, O) for the methyl and carboxyl groups and to to 1.2*U*_eq_(C) for the CH-groups. As the structure contains only light atoms,   Friedel pairs were merged and the Flack parameter value has not been determined.

### Crystal data

**Table:**

C_12_H_11_NO_4_	*F*(000) = 488
*M**_r_* = 233.2	*D*_x_ = 1.433 Mg m^−^^3^
Monoclinic, *P*2_1_	Cu *K*α radiation, λ = 1.5418 Å
Hall symbol: P 2yb	Cell parameters from 10329 reflections
*a* = 3.8564 (5) Å	θ = 3.2–67.0°
*b* = 27.178 (3) Å	µ = 0.92 mm^−^^1^
*c* = 10.4681 (9) Å	*T* = 120 K
β = 99.966 (9)°	Plate, yellow
*V* = 1080.6 (2) Å^3^	0.57 × 0.15 × 0.05 mm
*Z* = 4	

### Data collection

**Table:**

Agilent Xcalibur diffractometer with an Atlas (Gemini ultra Cu) detector	1922 independent reflections
Radiation source: Enhance Ultra (Cu) X-ray Source	1638 reflections with *I* > 3σ(*I*)
mirror	*R*_int_ = 0.083
Detector resolution: 10.3784 pixels mm^-1^	θ_max_ = 67.2°, θ_min_ = 3.3°
Rotation method data acquisition using ω scans	*h* = −4→4
Absorption correction: multi-scan (*CrysAlis PRO*; Agilent, 2010)	*k* = −32→32
*T*_min_ = 0.525, *T*_max_ = 1	*l* = −12→12
17777 measured reflections	

### Refinement

**Table:**

Refinement on *F*^2^	83 constraints
*R*[*F*^2^ > 2σ(*F*^2^)] = 0.049	H atoms treated by a mixture of independent and constrained refinement
*wR*(*F*^2^) = 0.129	Weighting scheme based on measured s.u.'s *w* = 1/[σ^2^(*I*) + 0.0025000002*I*^2^]
*S* = 1.73	(Δ/σ)_max_ = 0.005
1922 reflections	Δρ_max_ = 0.23 e Å^−^^3^
314 parameters	Δρ_min_ = −0.23 e Å^−^^3^
2 restraints	

### Special details

**Table:**

Experimental. CrysAlisPro (Agilent Technologies, 2010) Empirical absorption correction using spherical harmonics, implemented in SCALE3 ABSPACK scaling algorithm.
Refinement. The refinement was carried out against all reflections. The conventional *R*-factor is always based on *F*. The goodness of fit as well as the weighted *R*-factor are based on *F* and *F*^2^ for refinement carried out on *F* and *F*^2^, respectively. The threshold expression is used only for calculating *R*-factors *etc*. and it is not relevant to the choice of reflections for refinement.The program used for refinement, Jana2006, uses the weighting scheme based on the experimental expectations, see _refine_ls_weighting_details, that does not force *S* to be one. Therefore the values of *S* are usually larger than the ones from the *SHELX* program.The crystal studied was a non-merohedral twin with a minor twin domain of 24.7 (8)%. The overlaps of reflection between the twin domains were calculated by Jana2006 software using the twinning matrix and user- defined thresholds 0.23° for full overlap and 0.35° for full separation. For fully overlapped reflections, only a partial *F*^2^, corresponding to the twin volume fraction, were used in the refinement. Partially overlapped reflections were discarded from the refinement.

### Fractional atomic coordinates and isotropic or equivalent isotropic displacement parameters (Å^2^)

**Table:**

	*x*	*y*	*z*	*U*_iso_*/*U*_eq_	
O1	0.2649 (11)	0.6335 (11)	0.4130 (3)	0.0326 (11)	
O2	0.4957 (11)	0.5846 (11)	0.2764 (3)	0.0345 (12)	
O3	−0.0165 (9)	0.5839 (11)	0.7938 (3)	0.0283 (10)	
O4	−0.1254 (10)	0.5233 (11)	0.9851 (3)	0.0308 (11)	
O5	0.5833 (10)	0.6646 (11)	1.1452 (3)	0.0320 (12)	
O6	0.3472 (10)	0.7133 (11)	1.2816 (3)	0.0332 (12)	
O7	0.8594 (9)	0.7170 (11)	0.7627 (3)	0.0283 (10)	
O8	0.9534 (10)	0.7793 (11)	0.5719 (3)	0.0342 (12)	
N1	0.5464 (13)	0.4678 (11)	0.3684 (4)	0.0343 (14)	
N2	0.2837 (13)	0.8295 (11)	1.1899 (4)	0.0321 (14)	
C1	0.1528 (12)	0.5171 (11)	0.6702 (5)	0.0236 (14)	
C2	0.0453 (13)	0.5351 (11)	0.7835 (4)	0.0236 (14)	
C3	−0.0160 (12)	0.5019 (11)	0.8808 (4)	0.0245 (14)	
C4	0.0298 (13)	0.4524 (11)	0.8650 (4)	0.0255 (14)	
C5	0.1332 (14)	0.4346 (11)	0.7525 (5)	0.0288 (15)	
C6	0.1917 (13)	0.4663 (11)	0.6560 (4)	0.0274 (15)	
C7	0.2100 (13)	0.5534 (11)	0.5742 (4)	0.0250 (14)	
C8	0.3328 (14)	0.5483 (11)	0.4605 (4)	0.0243 (14)	
C9	0.4445 (14)	0.5034 (11)	0.4110 (4)	0.0277 (14)	
C10	0.3636 (13)	0.5926 (11)	0.3818 (4)	0.0257 (14)	
C11	0.1964 (15)	0.6094 (11)	0.9025 (5)	0.0332 (16)	
C12	−0.1818 (14)	0.4919 (11)	1.0900 (5)	0.0334 (16)	
C13	0.6801 (13)	0.7825 (11)	0.8888 (4)	0.0234 (13)	
C14	0.7893 (13)	0.7657 (11)	0.7738 (4)	0.0247 (14)	
C15	0.8358 (13)	0.7994 (11)	0.6769 (4)	0.0283 (15)	
C16	0.7721 (14)	0.8488 (11)	0.6932 (5)	0.0289 (15)	
C17	0.6679 (14)	0.8651 (11)	0.8067 (5)	0.0286 (15)	
C18	0.6238 (13)	0.8332 (11)	0.9037 (4)	0.0273 (15)	
C19	0.6324 (13)	0.7453 (11)	0.9840 (4)	0.0229 (14)	
C20	0.5098 (13)	0.7499 (11)	1.0980 (4)	0.0250 (14)	
C21	0.3867 (13)	0.7943 (11)	1.1482 (4)	0.0249 (14)	
C22	0.4825 (13)	0.7055 (11)	1.1777 (4)	0.0255 (14)	
C23	0.6528 (14)	0.6915 (11)	0.6539 (5)	0.0311 (15)	
C24	0.9902 (15)	0.8114 (11)	0.4668 (5)	0.0357 (17)	
H4	−0.00949	0.429884	0.931765	0.0306*	
H5	0.164009	0.399816	0.742312	0.0346*	
H6	0.259432	0.453504	0.578406	0.0329*	
H7	0.151405	0.586516	0.594259	0.03*	
H11a	0.27646	0.640183	0.873092	0.0498*	
H11b	0.05723	0.615435	0.968529	0.0498*	
H11c	0.395751	0.589449	0.937303	0.0498*	
H12a	−0.277146	0.510825	1.153105	0.0501*	
H12b	−0.343934	0.466212	1.057065	0.0501*	
H12c	0.038147	0.47757	1.129784	0.0501*	
H16	0.799855	0.871938	0.626328	0.0347*	
H17	0.62584	0.899564	0.817253	0.0344*	
H18	0.554605	0.845365	0.981646	0.0327*	
H19	0.697052	0.712524	0.963476	0.0275*	
H23a	0.668317	0.656625	0.668973	0.0466*	
H23b	0.411361	0.70166	0.644608	0.0466*	
H23c	0.741702	0.699178	0.576118	0.0466*	
H24a	1.067113	0.792805	0.398881	0.0535*	
H24b	0.767556	0.826519	0.434114	0.0535*	
H24c	1.160734	0.836408	0.496816	0.0535*	
H6o	0.315 (19)	0.6834 (14)	1.301 (7)	0.0498*	
H2o	0.50 (2)	0.6115 (17)	0.235 (6)	0.0517*	

### Atomic displacement parameters (Å^2^)

**Table:**

	*U*^11^	*U*^22^	*U*^33^	*U*^12^	*U*^13^	*U*^23^
O1	0.051 (2)	0.0260 (17)	0.0237 (16)	−0.0015 (15)	0.0152 (16)	0.0005 (13)
O2	0.053 (2)	0.0316 (19)	0.0222 (16)	−0.0003 (18)	0.0166 (16)	0.0036 (14)
O3	0.039 (2)	0.0282 (17)	0.0183 (14)	0.0052 (15)	0.0074 (14)	0.0006 (13)
O4	0.042 (2)	0.0356 (18)	0.0183 (15)	0.0008 (16)	0.0162 (15)	0.0033 (13)
O5	0.048 (2)	0.0256 (18)	0.0249 (18)	0.0029 (15)	0.0118 (16)	0.0038 (13)
O6	0.047 (2)	0.0329 (19)	0.0238 (16)	0.0009 (17)	0.0181 (16)	0.0038 (14)
O7	0.037 (2)	0.0304 (18)	0.0177 (14)	0.0057 (14)	0.0058 (14)	−0.0018 (13)
O8	0.046 (2)	0.042 (2)	0.0183 (16)	0.0056 (18)	0.0151 (16)	0.0045 (14)
N1	0.046 (3)	0.036 (2)	0.0238 (19)	0.000 (2)	0.0145 (19)	−0.0008 (17)
N2	0.041 (3)	0.032 (2)	0.026 (2)	−0.0008 (19)	0.0129 (19)	−0.0004 (17)
C1	0.022 (2)	0.029 (2)	0.021 (2)	0.0003 (19)	0.0066 (18)	0.0038 (18)
C2	0.022 (2)	0.028 (2)	0.021 (2)	0.0016 (18)	0.0043 (19)	0.0002 (18)
C3	0.023 (2)	0.036 (2)	0.017 (2)	0.0012 (19)	0.0094 (18)	0.0015 (19)
C4	0.026 (2)	0.031 (2)	0.020 (2)	−0.0031 (19)	0.0053 (19)	0.0032 (17)
C5	0.035 (3)	0.029 (2)	0.023 (2)	−0.002 (2)	0.008 (2)	0.0023 (18)
C6	0.032 (3)	0.030 (2)	0.022 (2)	−0.001 (2)	0.009 (2)	−0.0003 (19)
C7	0.028 (3)	0.028 (2)	0.020 (2)	−0.0008 (19)	0.0069 (19)	0.0026 (18)
C8	0.028 (3)	0.027 (2)	0.018 (2)	−0.0036 (19)	0.0045 (19)	−0.0008 (17)
C9	0.035 (3)	0.028 (2)	0.020 (2)	−0.004 (2)	0.007 (2)	0.0008 (18)
C10	0.030 (3)	0.030 (3)	0.018 (2)	−0.0027 (19)	0.0073 (19)	0.0007 (18)
C11	0.040 (3)	0.034 (3)	0.027 (2)	0.001 (2)	0.010 (2)	−0.0020 (19)
C12	0.037 (3)	0.045 (3)	0.020 (2)	0.003 (2)	0.012 (2)	0.008 (2)
C13	0.026 (3)	0.028 (2)	0.0164 (19)	−0.0014 (19)	0.0057 (19)	0.0029 (17)
C14	0.028 (3)	0.028 (2)	0.019 (2)	0.002 (2)	0.0063 (19)	0.0017 (18)
C15	0.029 (3)	0.037 (3)	0.020 (2)	−0.002 (2)	0.008 (2)	0.001 (2)
C16	0.032 (3)	0.033 (3)	0.023 (2)	−0.002 (2)	0.007 (2)	0.0092 (18)
C17	0.033 (3)	0.026 (2)	0.028 (2)	−0.002 (2)	0.008 (2)	0.0022 (19)
C18	0.032 (3)	0.030 (2)	0.020 (2)	0.000 (2)	0.005 (2)	−0.0018 (18)
C19	0.025 (3)	0.025 (2)	0.019 (2)	−0.0012 (19)	0.0045 (19)	0.0013 (18)
C20	0.026 (2)	0.027 (2)	0.022 (2)	−0.0017 (19)	0.005 (2)	0.0033 (18)
C21	0.031 (3)	0.027 (2)	0.0177 (19)	−0.0022 (19)	0.0084 (19)	0.0035 (17)
C22	0.030 (3)	0.026 (2)	0.022 (2)	−0.0027 (19)	0.006 (2)	0.0013 (18)
C23	0.035 (3)	0.032 (3)	0.026 (2)	−0.002 (2)	0.005 (2)	−0.0026 (19)
C24	0.039 (3)	0.049 (3)	0.024 (2)	−0.002 (3)	0.016 (2)	0.007 (2)

### Geometric parameters (Å, °)

**Table:**

O1—C10	1.24 (4)	C8—C9	1.42 (4)
O2—C10	1.311 (9)	C8—C10	1.48 (3)
O2—H2o	0.85 (6)	C11—H11a	0.96
O3—C2	1.35 (4)	C11—H11b	0.96
O3—C11	1.46 (2)	C11—H11c	0.96
O4—C3	1.367 (18)	C12—H12a	0.96
O4—C12	1.44 (2)	C12—H12b	0.96
O5—C22	1.24 (4)	C12—H12c	0.96
O6—C22	1.303 (9)	C13—C14	1.418 (15)
O6—H6o	0.85 (5)	C13—C18	1.41 (4)
O7—C14	1.36 (4)	C13—C19	1.45 (3)
O7—C23	1.45 (2)	C14—C15	1.40 (3)
O8—C15	1.373 (17)	C15—C16	1.38 (4)
O8—C24	1.43 (3)	C16—C17	1.391 (15)
N1—C9	1.16 (3)	C16—H16	0.96
N2—C21	1.15 (3)	C17—C18	1.37 (3)
C1—C2	1.411 (16)	C17—H17	0.96
C1—C6	1.40 (4)	C18—H18	0.96
C1—C7	1.45 (3)	C19—C20	1.364 (8)
C2—C3	1.41 (3)	C19—H19	0.96
C3—C4	1.37 (4)	C20—C21	1.43 (3)
C4—C5	1.394 (16)	C20—C22	1.48 (3)
C4—H4	0.96	C23—H23a	0.96
C5—C6	1.38 (3)	C23—H23b	0.96
C5—H5	0.96	C23—H23c	0.96
C6—H6	0.96	C24—H24a	0.96
C7—C8	1.362 (8)	C24—H24b	0.96
C7—H7	0.96	C24—H24c	0.96
			
C10—O2—H2o	109 (5)	H12a—C12—H12b	109.4706
C2—O3—C11	116.4 (14)	H12a—C12—H12c	109.4709
C3—O4—C12	118 (2)	H12b—C12—H12c	109.4715
C22—O6—H6o	98 (5)	C14—C13—C18	118.8 (17)
C14—O7—C23	116.3 (14)	C14—C13—C19	117 (2)
C15—O8—C24	118 (2)	C18—C13—C19	124.4 (11)
C2—C1—C6	119.1 (17)	O7—C14—C13	118.5 (17)
C2—C1—C7	117 (2)	O7—C14—C15	121.5 (12)
C6—C1—C7	124.4 (12)	C13—C14—C15	120 (2)
O3—C2—C1	119.3 (17)	O8—C15—C14	115 (2)
O3—C2—C3	121.0 (12)	O8—C15—C16	125.2 (18)
C1—C2—C3	120 (2)	C14—C15—C16	119.9 (12)
O4—C3—C2	115 (2)	C15—C16—C17	119.9 (18)
O4—C3—C4	125.3 (17)	C15—C16—H16	120.0398
C2—C3—C4	120.0 (12)	C17—C16—H16	120.0393
C3—C4—C5	120.3 (18)	C16—C17—C18	122 (3)
C3—C4—H4	119.845	C16—C17—H17	119.1935
C5—C4—H4	119.8442	C18—C17—H17	119.193
C4—C5—C6	121 (3)	C13—C18—C17	119.8 (13)
C4—C5—H5	119.6762	C13—C18—H18	120.1038
C6—C5—H5	119.6755	C17—C18—H18	120.1024
C1—C6—C5	120.4 (13)	C13—C19—C20	130 (2)
C1—C6—H6	119.8163	C13—C19—H19	115.0573
C5—C6—H6	119.8174	C20—C19—H19	115.0579
C1—C7—C8	131 (2)	C19—C20—C21	126 (2)
C1—C7—H7	114.6249	C19—C20—C22	119 (2)
C8—C7—H7	114.6246	C21—C20—C22	114.9 (9)
C7—C8—C9	125 (2)	N2—C21—C20	178.7 (18)
C7—C8—C10	119 (2)	O5—C22—O6	124 (2)
C9—C8—C10	116.0 (9)	O5—C22—C20	121.2 (9)
N1—C9—C8	177.1 (17)	O6—C22—C20	115 (2)
O1—C10—O2	124 (2)	O7—C23—H23a	109.4707
O1—C10—C8	121.9 (9)	O7—C23—H23b	109.4723
O2—C10—C8	115 (2)	O7—C23—H23c	109.472
O3—C11—H11a	109.4714	H23a—C23—H23b	109.4696
O3—C11—H11b	109.4709	H23a—C23—H23c	109.4705
O3—C11—H11c	109.4705	H23b—C23—H23c	109.4722
H11a—C11—H11b	109.4718	O8—C24—H24a	109.4715
H11a—C11—H11c	109.4715	O8—C24—H24b	109.4709
H11b—C11—H11c	109.4713	O8—C24—H24c	109.4717
O4—C12—H12a	109.471	H24a—C24—H24b	109.4707
O4—C12—H12b	109.4717	H24a—C24—H24c	109.4706
O4—C12—H12c	109.4716	H24b—C24—H24c	109.4718
			
C1—C2—O3—C11	−120.5 (7)	C13—C14—O7—C23	121.0 (7)
C2—C3—O4—C12	−178.0 (5)	C14—C15—O8—C24	177.1 (6)

### Hydrogen-bond geometry (Å, °)

**Table:**

*D*—H···*A*	*D*—H	H···*A*	*D*···*A*	*D*—H···*A*
O6—H6o···O1^i^	0.85 (5)	1.82 (6)	2.62 (3)	154 (6)
O2—H2o···O5^ii^	0.85 (6)	1.79 (6)	2.63 (3)	169 (8)
C11—H11c···O4^iii^	0.96	2.56	3.50 (3)	167.
C23—H23a···O3^iii^	0.96	2.56	3.42 (4)	149.

Symmetry codes: (i) *x*, *y*, *z*+1; (ii) *x*, *y*, *z*−1; (iii) *x*+1, *y*, *z*.
